# A Novel Asymmetric Diffusion Path for Superior Ion Dynamic in High‐Voltage Mg‐Based Hybrid Batteries

**DOI:** 10.1002/advs.202406451

**Published:** 2024-09-04

**Authors:** Kaifeng Huang, Baihua Qu, Xing Shen, Rongrui Deng, Rong Li, Guangsheng Huang, Aitao Tang, Qian Li, Jingfeng Wang, Fusheng Pan

**Affiliations:** ^1^ College of Materials Science and Engineering National Engineering Research Center for Magnesium Alloys Chongqing University Chongqing 400044 China; ^2^ Chongqing Institute of New Energy Storage Materials and Equipment Chongqing 401135 China

**Keywords:** asymmetric diffusion path, diffusion kinetics, dual‐salt electrolyte, high‐voltage cathode, Mg‐based hybrid batteries

## Abstract

Magnesium‐based batteries have garnered significant attention due to their high energy density, excellent intrinsic safety, and low cost. However, the application process has been hindered by the high Mg^2+^ ions diffusion barrier in solid‐state structures and solid‐liquid interphase. To address this issue, a hybrid battery technology based on Mg anode and Fe‐based Prussian Blue Analogue cathode doped with functional transition metal ions and N═O bonds is proposed. Combined multiscale experimental characterizations with theoretical calculations, the subtle lattice distortion can create an asymmetric diffusion path for the active ions, which enables reversible extraction with significantly reduced diffusion barriers achieved by synergistic doping. The optimized cathode exhibits a working potential of 2.3 V and an initial discharge capacity of 152 mAh g^−1^ at 50 mA g^−1^. With the preferred electrolyte combined with equivalent concentration [Mg_2_(µ‐Cl)_2_(DME)_4_][AlCl_4_]_2_ and NaTFSI salt solution, the hybrid system demonstrates superior cycling performance over 200 cycles at a high current density of 200 mA g^−1^, maintaining ≈100% coulombic efficiency with superior ion dynamic. The findings are expected to be marked an important step in the further application of high‐voltage cathodes for Mg‐based hybrid batteries.

## Graphical abstract

To obtain a Mg‐base hybrid battery with good dynamic performance, TFSI^−^ ions are used as electrolyte additives to improve the electrochemical window and shorten the activation process. Meanwhile, an asymmetric diffusion path is created in the cathode structure by codoping of cations and N═O bond to achieve the superior Mg/Na ion storage electrochemical performance.

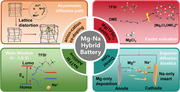

## 1 Introduction

Driven by climate change and environmental concerns, the development of clean energy has been a major focus in recent years. Lithium‐ion batteries (LIBs) have spearheaded energy storage applications for decades owing to high energy density and mature manufacturing technology,^[^ ^1^, ^2^, ^3^ ^]^ but the limited resource reserves and wildly fluctuating prices trigger the exploitation of alternative energy storage systems.^[^ ^4^, ^5^ ^]^ In response to the need for energy storage, Na‐ion batteries (NIBs) stand out as a feasible substitute due to their wide availability and cost‐effectiveness.^[^ ^6^, ^7^, ^8^ ^]^ Still, they also face challenges such as dendrite‐induced safety problems and imperfect energy density when matched with carbon‐based anode materials.^[^ ^9^, ^10^, ^11^ ^]^ Recently, Magnesium‐based batteries have drawn attention as a novel renewable energy storage solution for their ample resources, competitive costs, and dendrite‐free deposition.^[^ ^12^, ^13^, ^14^, ^15^, ^16^ ^]^ However, the large charge/radius ratio and high polarization of the Mg^2+^ ions lead to strong electrostatic interactions between Mg^2+^ cations and the anions in the lattice, which greatly retard the diffusion kinetics of the Mg^2+^ ions.^[^ ^17^, ^18^, ^19^, ^20^, ^21^ ^]^ To overcome these hurdles, Mg‐Na hybrid batteries (MNHBs) have been developed to take full use of their advantages and avoid defects as far as possible.^[^ ^22^, ^23^, ^24^, ^25^, ^26^, ^27^, ^28^, ^29^ ^]^ This approach uses Mg metal as the anode to enhance safety, and reversible de‐intercalation of Na^+^ ions in the cathode is facilitated due to their better mobility within solid structures compared to the multivalent Mg^2+^ ions. By the integration of an Mg‐Na dual salt electrolyte, the hybrid battery system was established with decent energy density and inherent safety.^[^ ^30^, ^31^ ^]^

So far, varieties of MNHBs have been explored to harness the combined advantages of Na^+^ and Mg^2+^ ions in electrochemical processes, especially for cathode materials and electrolyte regulation. Due to the electrolyte‐determining solvation structures of metal ions, which further affect diffusion mode in cathode materials, a well‐designed system of high‐performance cathodes and compatible electrolytes is essential. For instance, Dong et al.^[^ ^32^ ^]^ employed high‐voltage FeFe(CN)_6_ as cathode material and chlorine‐based species as the hybrid electrolyte in MNHBs, revealing the reaction mechanism features of Daniel‐type hybrid batteries that Mg^2+^ ions are usually deposited/dissolved on the Mg metal anode, while only Na^+^ ions are inserted/deinserted into the cathode.^[^ ^33^ ^]^ Due to the short diffusion distance for the Daniel‐type hybrid battery, it shows outstanding diffusion kinetics in cathode materials and electrolyte solution. Bian et al.^[^ ^34^ ^]^ used TiS_2_ as the cathode material in MNHBs, demonstrating co‐intercalation type in a chlorine‐free electrolyte system. Here, Mg^2+^ and Na^+^ ions both insert into the cathode materials to achieve superior capacity. Cabello et al.^[^ ^35^ ^]^ reported NaV_6_O_15_ as a cathode and chlorine‐free electrolyte to form a Rocking‐chair hybrid battery, in which both Mg^2+^ and Na^+^ ions are involving the electrochemical reaction process at the cathode and anode. However, the Na^+^ deposition on the Mg anode is likely to result in the formation of dendrite, thereby causing a decline in cycling performance with poor safety. Thus, it is imperative to rationalize the match of dual‐salt electrolyte and cathode material, which exhibits unique electrochemical behavior and functional properties.

**Scheme 1:**
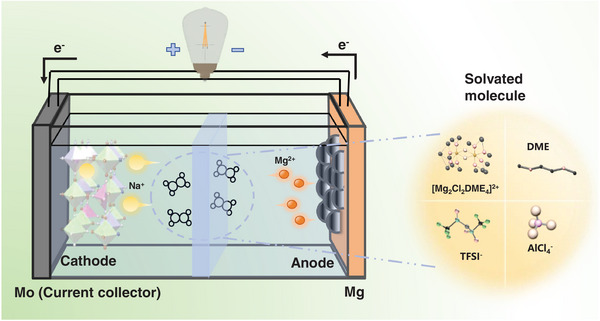
The schematic diagram of Mg‐Na hybrid batteries based on ZMNO‐PBA cathode and high‐voltage dual‐salts electrolyte.

Herein, we propose a Daniel‐type hybrid battery with a novel anion‐cation co‐doping cathode Zn_0.3_Mn_0.15_Fe(CN)_5_NO (ZMNO‐PBA) by utilizing sodium nitroprusside (Na_2_Fe(CN)_5_NO) as the N═O introducer and employ hybrid salts containing NaTFSI and Mg(TFSI)_2_ as the electrolyte, as shown in **Scheme** **1**. The modified cathode preserved the original face‐centered cubic crystal structure but induced unique trace structural distortions with the introduction of N═O bonds compared with Zn_0.46_Mn_1.29_[Fe(CN)_6_] (ZM‐PBA) (Figure S1, Supporting Information). The ZMNO‐PBA cathode demonstrated a reversible discharge capacity of 126 mAh g^−1^ at 50 mA g^−1^ and maintained a Coulombic efficiency of about 98% over 100 cycles at 200 mA g^−1^. Besides, the novel hybrid electrolyte compositions with enhanced voltage window up to 3.9 V and improved impurities tolerance were designed. The MNHB system is composed of appropriate electrolytes and the optimized ZMNO‐PBA cathode shows a specific energy of 289.9 Wh kg^−1^ at 50 mA g^−1^_._

## 2 Results and Discussion

### 2.1 Electrolyte Properties

To develop MNHBs with excellent electrochemical performance, a crucial step involves designing electrolytes with low overpotentials, wide electrochemical window, and good compatibility with Mg^2+^ and Na^+^ ions. Common Mg‐Na hybrid electrolytes include Mg(BH_4_)_2_+Na(BH_4_)^[^ ^34^, ^35^, ^36^ ^]^ and [Mg_2_Cl_2_][AlCl_4_]_2_+NaAlCl_4_,^[^ ^37^, ^38^ ^]^showing decent performance in hybrid Mg‐Na systems. While, the former has noncorrosiveness with low anodic stability and poor Columbic efficiency, and the latter has higher anodic stability but concerning the side reaction in Equation (1)

(1) $$\begin{array}{ccc} 4{\mathrm{AlCl}}_{4}^{-} & + & 6Mg_{\mathrm{metal}}+12\mathrm{DME}\to 3{\left[Mg_{2}{\left(\mu -\mathrm{Cl}\right)}_{2}{\left(\mathrm{DME}\right)}_{4}\right]}^{2+}\\ & & +\;4Al_{\mathrm{metal}}+10Cl^{-} \end{array}$$

This reaction requires high‐purity precursors, long electrochemical conditioning process, and suffers from excessive chloride ion side effects.^[^ ^39^ ^]^ To alleviate the chloride corrosion effects and enhance anodic stability, the [Mg_2_(µ‐Cl)_2_(DME)_4_][AlCl_4_]_2_+Mg(TFSI)_2_+NaTFSI (CNTT) hybrid electrolyte was employed. As reported, the addition of Mg(TFSI)_2_ could induce the transformation of free anions (Equation 2)^[^ ^40^ ^]^

(2) $$\begin{array}{c} \mathrm{MgC}l_{2}+\mathrm{Mg}{\left(\mathrm{TFSI}\right)}_{2}+4\mathrm{DME}\to {\left[Mg_{2}{\left(\mu -\mathrm{Cl}\right)}_{2}{\left(\mathrm{DME}\right)}_{4}\right]}^{2+}+2\mathrm{TFS}I^{-} \end{array}$$

where the unique component design can facilitate the conversion of MgCl_2_ into electrochemically active species, and lead to an increased concentration of [Mg_2_(µ‐Cl)_2_(DME)_4_]^2+^ without electrochemical conditioning.^[^ ^41^ ^]^ As depicted in Figure S2a (Supporting Information), Linear Sweep Voltammetry (LSV) testing demonstrates that the anodic stability of the CNTT hybrid electrolyte is ≈3.9 V (vs Mg/Mg^2+^). The cyclic voltammetry (CV) test for the initial cycle of the hybrid electrolyte is shown in Figure S2b (Supporting Information), which demonstrates good stripping/deposition behaviors of Mg species on Mo foil. The Mg|CNTT|Mg symmetric cell (Figure S2c, Supporting Information) exhibits an overpotential of about 160 mV at 0.1 mA cm^−2^ after 400 h. Consequently, as seen in Figure S3a,b (Supporting Information), the deposition layer around the center of the Mg anode becomes thicker than the layers on either side, with a considerable amount of sediment accumulating at the center regions. Moreover, EDX Mapping (Figure S3c–f, Supporting Information) analysis revealed evidence of Mg‐only deposition on the anode. In the hybrid ion system, disparities in diffusion rates between Na^+^ and Mg^2+^ ions lead to a situation where Mg^2+^ ions fail to replenish into the electrolyte promptly when Na^+^ ions are embedded in the cathode material. This causes concentration polarization and leads to deviation from equilibrium potential during the discharge process. Therefore, the CNTT electrolyte accelerates the solvation process of Mg^2+^ ions, thereby achieving a good match with Na^+^ ions diffusion, resulting in a stable hybrid battery with superior dynamics.

### 2.2 Structural Characterizations

**Figure 1:**
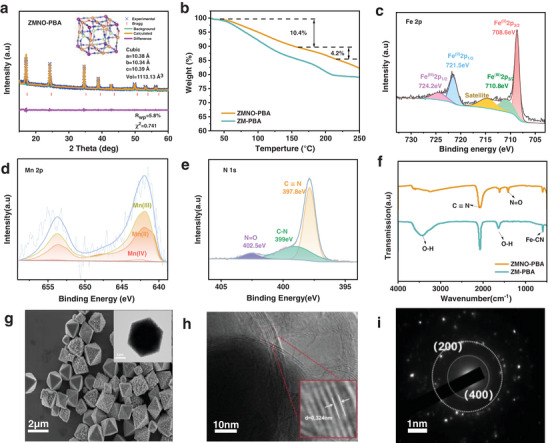
Structural and morphology characterization. a) Rietveld refinement of ZMNO‐PBA. b) TG curves of ZMNO‐PBA and ZM‐PBA. XPS spectra of ZMNO‐PBA: c) Fe 2p; d) Mn 2p; e) N 1s. f) FTIR spectra of ZMNO‐PBA and ZM‐PBA. g) SEM image of ZMNO‐PBA material with TEM image as insets. h) High‐resolution TEM images with the enlarged region as insets and i) SAED pattern of ZMNO‐PBA.

In addition to the rational design of dual‐salt electrolytes, the favorable selection of well‐matched cathode materials is critical to attaining high energy density and a long cycling period. Prussian blue analogs (PBA)^[^ ^42^, ^43^, ^44^ ^]^ have an open structure and high voltage characteristics, thus offering the possibility to satisfy these requirements. ZMNO‐PBA was synthesized by a simple hydrothermal method.^[^ ^45^ ^]^ The ICP analysis (Table S1, Supporting Information) reveals that the ratio of Zn and Mn in ZMNO‐PBA, which is significantly lower compared to ZM‐PBA, indicating the transition metal elements in ZMNO are mainly Fe. **Figures** **1**a and S4 (Supporting Information) show the Rietveld refinement results of XRD patterns, which manifests that the crystal structures of ZMNO‐PBA and ZM‐PBA are well indexed to the face‐centered cubic structure with the space group of F m – 3 m. The lattice volume of ZMNO‐PBA is calculated as 1113.13 Å^3^, which is smaller than the ZM‐PBA sample. All the diffraction peaks of ZMNO‐PBA shift to a low degree, representing a larger crystal spacing compared to the conventional Prussian blue analogue Fe_4_[Fe(CN)_6_]_3_. Thermogravimetric analysis (TG), depicted in Figure 1b, was employed to assess the water content within both structures. Within materials, structural water can be classified into two categories: adsorbed water and interstitial water, the mass loss within the temperature range of 30–150 °C corresponds to the adsorbed water, whereas the mass decrement between 150–220 °C signifies the quantity of interstitial water.^[^ ^46^, ^47^ ^]^ From these evaluations, ZMNO‐PBA exhibits notably lower levels of both adsorbed and interstitial water when compared to ZM‐PBA, implying that ZMNO‐PBA possesses more Na^+^ storage sites which is beneficial to release higher capacity. Besides, the valence states of Fe and Mn in the ZMNO‐PBA samples were detected by X‐ray photoelectron spectroscopy (XPS) and the results are shown in Figure 1c,d, respectively. The ratio of Fe (II): Fe (III) is determined to be 0.55:0.45, suggesting that the mean valence state of the low‐spin state Fe is ≈2.6. Furthermore, the Mn (II):Mn (III):Mn (IV) ratio stands at 0.40:0.57:0.018, indicating that the average valence state of Mn is about 2.72. The absorption peak located at 402.5 eV for N 1s spectra (Figure 1e) confirmed the presence of N═O bonding effects, however, there is no absorption peak corresponding to the N═O bond in ZM‐PBA^[^ ^45^ ^]^ (Figure S5, Supporting Information). Meanwhile, the absorption peaks located at 599.4, 1414.4, and 2071.7 cm^−1^ in the FTIR spectra (Figure 1f) correspond to vibrations of Fe–CN, N═O, and C≡N bonds, respectively, which further demonstrates that the metal‐organic frameworks are composed of N═O and C≡N bonds. Besides, the absorption peaks at 3248.9 and 1611.3 cm^−1^ for the O–H bonds can also reflect that the ZMNO‐ PBA structure contains less structural water.

The SEM and TEM image in Figure 1g shows the regular octahedra morphology of ZMNO‐PBA, which is due to the positive effect of Zn doping with a slow coordination rate. It can be seen that the particle size of ZMNO‐PBA is between 2 and 3 µm under the influence of an efficient chelating agent, and they are stacked by primary nanoparticles. Except for regular octahedra particles, the slower coordination speed leads to partial particles failing to form the regular octahedra, resulting in the uneven surface with improved surface energy, which could contribute to strengthened ions absorption and significantly enhance the charge–discharge capacity. In contrast, Figure S6a (Supporting Information) shows that MnFe(CN)_5_NO without Zn doping displays a regular cubic morphology, whereas ZM‐PBA (Figure S6b–i, Supporting Information) exhibits an irregular agglomeration of particles with a particle size of ≈1–2 µm. The high‐resolution TEM in Figure 1h illustrates the interplanar spacing of the (311) crystalline face with 0.324 nm for the ZMNO‐PBA cathode. Also, the SAED image in Figure 1i shows the characteristic polycrystalline diffraction ring with the observed (200) and (400) crystal faces.

### 2.3 Electrochemical Properties

**Figure 2:**
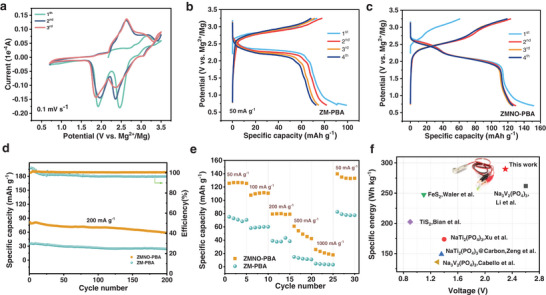
Electrochemical Properties. a) CV curves of ZMNO‐PBA at a scan rate of 0.1 mV s^−1^. Charge and discharge profiles of the b) ZM‐PBA and c) ZMNO‐PBA at 50 mA g^−1^. d) Long‐term cycle performance of ZM‐PBA and ZMNO‐PBA cathodes at 200 mA g^−1^. e) Rate performance of ZM‐PBA and ZMNO‐PBA cathodes. f) The specific energy‐voltage plots for typical Mg–Na hybrid systems with the electronic picture of a green LED lamp lightened by a Mg–Na hybrid coin cell as insets.

The electrochemical behavior of ZMNO‐PBA was evaluated by CV test at a scan rate of 0.1 mV s^−1^. As shown in **Figure** **2**a. Two pairs of redox peaks can be detected at 3.0/2.5, and 2.4/1.9 V, which correspond to the Mn^3+^/Mn^2+^ and Fe^3+^/Fe^2+^ redox couples, respectively. The XPS analysis conducted post‐initial discharge of ZMNO‐PBA (Figure S7, Supporting Information) further substantiates the involvement of Mn and Fe in redox reactions. Specifically, the ratio of Mn oxidation states after discharge is found to be Mn (II): Mn (III): Mn (IV) = 0.72:0.12:0.1(an average valence state of 2.5). Meanwhile, for Fe, the ratio of Fe (II): Fe (III) = 0.72:0.28, which corresponds to an average valence of 2.28. These results offer a clear indication of the oxidation and reduction processes undergone by Mn and Fe within the ZMNO‐PBA material during the discharge cycle. While ZM‐PBA demonstrates the only distinctive characteristic redox peak at 3.0/1.9 V, indicating the inadequate electrochemical reaction of the Mn^3+^/Mn^2+^ couple (Figure S8, Supporting Information). The galvanostatic charge–discharge curves of the samples at 50 mA g^−1^ are presented in Figure 2b,c. ZMNO‐PBA exhibits a remarkable specific capacity of 152 mAh g^−1^ with high discharge plateaus centered at 2.5 and 1.9 V, whereas it is only 70 mAh g^−1^ for ZM‐PBA at the same current density. The long‐term structural durability of the samples was conducted under the current density of 200 mA g^−1^ in Figure 2d. As seen, the ZMNO‐PBA cathode shows capacity retention of 74.6% and a discharge capacity of 58.7 mAh g^−1^ after 200 cycles and the Coulombic efficiency remains ≈100%, while the ZM‐PBA displays unstable Coulombic efficiency(96%) and low reversible capacity of 24.26 mAh g^−1^ over 200cycles, with the capacity retentions to 66.6%. This may be due to the fact that the lattice structure with the N═O bond is more stable and exhibits better diffusion kinetics. Figure 2e showcases the rate performance for as‐prepared samples, and the discharge capacities are 126, 110, 80, 45, and 20 mAh g^−1^ at the current density of 50, 100, 200, 500, and 1000 mA g^−1^ for ZMNO‐PBA, respectively. And it can be found that the specific discharge capacity is significantly improved when the current density returns to 50 mA g^−1^, which is because the material reaches a larger specific discharge capacity after a certain cyclic activation process.^[^ ^48^, ^49^, ^50^ ^]^ Due to the strong Coulombic interaction of Mg^2+^ ions, their diffusion rate is relatively slow, leading to a substantial decrease in specific capacity when tested at high current density. Furthermore, with the increasing current densities (Figure S8b, Supporting Information), the discharging plateau gradually degrades, Additionally, through CV tests conducted at various scan rates (Figure S8c,d, Supporting Information), implying that the discharge process is predominantly governed by diffusion‐controlled mechanisms, indicative of ion transport limitations within the electrode materials. Conversely, the charging process is mainly controlled by pseudocapacitive behavior, suggesting rapid surface redox reactions that facilitate swift charge storage. Overall, ZMNO‐PBA presents a higher specific energy density (289.9 Wh kg^−1^), which is quite comparable to the typical reported Mg‐Na hybrid battery systems, furthermore, it could continuously power a green LED, as seen in Figure 2f, showcasing its promising practical applications.

**Figure 3:**
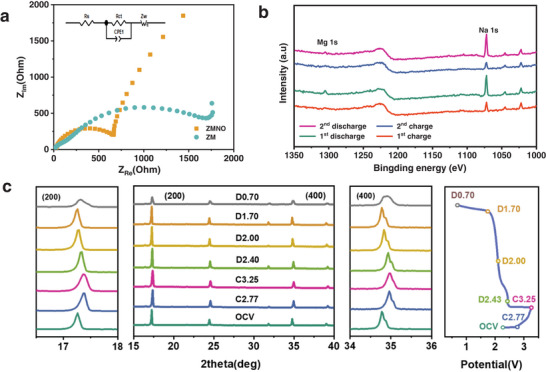
Diffusion mechanism and structural evolution. a) Nyquist plot of ZMNO‐PBA and ZM‐PBA. b) Ex situ XPS spectra and c) ex situ XRD patterns of ZMNO‐PBA at different charge–discharge states and the corresponding charge–discharge curves.

In order to investigate the diffusion capability and mechanism of hybrid ions. Nyquist plots (**Figure** **3**a) and GITT (Figure S9, Supporting Information) were subjected to attain a comparative analysis of ZMNO‐PBA and ZM‐PBA. The results showed that ZMNO‐PBA displays a lower charge transfer resistance profile. Specifically, the diffusive coefficients (*D*_diff_) of active ions in ZMNO‐PBA fluctuate between 10^−8^ to 10^−11^ cm^2^ s^−1^, whereas it ranges from 10^−9^ to 10^−12^ cm^2^ s^−1^ for ZM‐PBA, which suggests that the introduction of N═O functionalities into the ZMNO‐PBA structure could improve the diffusion kinetics. Ex‐situ XPS (Figure 3b) analysis was utilized to clarify the reaction mechanism for the MNHB. The appearance of the Mg 1s absorption peak during the initial discharge signifies the co‐intercalation of Mg^2+^ alongside Na^+^ within the cathode material. Nonetheless, the amount of Mg^2+^ intercalation is minimal, and they do not participate in the reversible de‐intercalation during the subsequent charge–discharge process. Instead, only Na^+^ undergo continuous insertion and extraction throughout the battery's operational life, which reveals a Daniel‐type ion diffusion mechanism. The ex situ X‐ray diffraction (Figure 3c) analysis demonstrates the extent of lattice change experienced by ZMNO‐PBA during the ion diffusion process. Following 4 h of infiltration with the electrolyte, the (200) and (400) reflection peaks show a progressive shift toward higher angles, and this trend persists during the charging stage. This observation indicates that the distance between these crystalline layers decreases once the Na^+^ is removed from the material. In contrast, the diffraction peaks of (200) and (400) returned to the opposite direction upon discharging, but they could not recover to the pristine state due to the irreversible microstructural distortion within the crystal lattice. This irreversible reduction in interplanar spacing is primarily attributed to the disparity in bond lengths between the N═O and C≡N groups in the ZMNO‐PBA structure. This distortion creates preferential diffusion channels that minimize the diffusion energy barrier for Na^+^, thereby affecting the reversibility of the intercalation/deintercalation process and influencing the long‐term stability and rate capability of the ZMNO‐PBA‐based battery system. In contrast, the reversible desodiation/sodiation process in ZM‐PBA (Figure S10, Supporting Information) also entails a variation of crystalline spacing, which expanded after the initial cycle. That is to say, the absorption peaks corresponding to the (200) and (400) crystal planes were observed to shift toward higher angles initially, and subsequently revert to their initial positions following the reinsertion of Na^+^. Throughout this entire process, the material experiences minor spatial distortions. These findings suggest that the C≡N structure within ZM‐PBA maintains its elasticity during the Na^+^ migration process, which ensures a high degree of symmetry and uniformity in the Na^+^ diffusion path.

### 2.4 Theoretical Calculation

**Figure 4:**
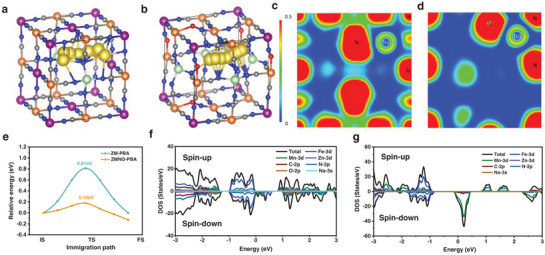
Theoretical Calculation. Diffusion path of a) ZM‐PBA and b) ZMNO‐PBA. Visualization of electron localization functions (ELF) of c) the (001) section of ZM‐PBA‐Na‐fs and d) the (100) section of ZMNO‐PBA‐Na‐fs. e) Diffusion barrier of Na^+^ in ZM‐PBA and ZMNO‐PBA. Density of states of f) ZMNO‐PBA and g) ZM‐PBA.

1.To further understand the diffusion kinetics features, density functional theory (DFT) analysis was applied with studying the structural variation during the electrochemical process. Based on the aforementioned Ex‐XPS data, it is evident that the system corresponds to a Daniel‐type battery. Therefore, in the subsequent calculation process, only Na^+^ would be taken into consideration for reversible de/intercalation. Here, Climbing Image Nudged Elastic Band (CI‐NEB) method was utilized to investigate the diffusion path of Na^+^ ions in the ZM‐PBA and ZMNO‐PBA structures (**Figures** **4**a,b and S11, Supporting Information). Since all samples exhibit cubic structures with high symmetry, the diffusion barriers along different paths and directions are identical. Similar to the aforementioned result of refined XRD, the ZM‐PBA structure exhibits less spatial distortion and possesses a highly symmetric Na^+^ diffusion path. In contrast, the introduction of N═O bonds leads to trace distortions in the crystal structure of ZMNO‐PBA and triggers the formation of an asymmetric diffusion path upon Na^+^ embedding. This is due to the differing bond strengths and bond lengths of the N═O and C≡N bonds, resulting in varying forces acting on the Na^+^ during embedding and leading to changes in diffusion paths. The slight spatial distortion in ZMNO‐PBA facilitates a more liberal movement of Na^+^ ions, effectively reducing the diffusion energy barrier of Na^+^ ions within the structure, all while maintaining better structural stability, which is consistent with the superior cycling stability. The Electron Localization Function (Figure 4c,d) calculation was also conducted in the Na‐embedded final‐state structure. As shown, ZMNO‐PBA manifests an asymmetric electron localization pattern, further substantiating the asymmetric configuration of the Na^+^ diffusion path within the structure. As shown in Figure 4e, the diffusion barrier for Na^+^ ions along the asymmetric diffusion path is 0.18 eV, which is much lower than that of the symmetric diffusion paths (0.81 eV) and further confirm that ZMNO‐PBA exhibits better diffusion kinetics. Moreover, the electronic properties after Na^+^ insertion were also analyzed by calculating the density of states (Figure 4f,g). Results show that both structures exhibit semi‐metallicity, illustrating the superior electronic conductivity of cation‐doped cathode, and the main orbital contributions around the Fermi level are influenced by the variation in substitutional metal content. Specifically, in ZM‐PBA, the metallicity mainly comes from the Mn element; while in ZMNO‐PBA, the metallicity mainly comes from the Fe‐3d spin‐down orbitals, which offer the largest contribution at the Fermi level. Meanwhile, the coordination environment and bonding characteristics of the C≡N bond and N═O bond in the outermost ZMNO‐PBA and ZM‐PBA surface are shown in COHP analyses (Figure S12, Supporting Information). Furthermore, the Integrated COHP (ICOHP) at the Fermi level for all occupied orbitals serves as a measure of the bonding strength: the more negative the ICOHP value, the stronger the bond. It is found that the C≡N in ZMNO‐PBA displays the most negative ICOHP (−1.1753) which illustrates the introducing N═O bond strengthened bonding interaction, enhancing the stability of ZMNO‐PBA structure. Thus, DFT calculation results illuminate the key factors underlying the favorable kinetic properties of asymmetric Na^+^ diffusion path and enhanced electronic conductivity with semi‐metallicity features, further confirm the structural regulation for cathode materials involving better rate/cycling capabilities and improved discharge capacity.

## 3 Conclusion

In summary, a low‐cost high‐voltage anion‐cation co‐doped ZMNO‐PBA cathode was successfully synthesized by using a facile hydrothermal method. The introduction of Zn^2+^, Mn^2+^ and N═O interactions effectively stabilizes the bulk structure with less interstitial water and induces preferable local lattice distortion to achieve better diffusion kinetics. Benefiting from the great structural features, the modified ZMNO‐PBA exhibits significantly enhanced specific capacity and superior rate capabilities compared to ZM‐PBA without N═O bonds. Under the current density of 200 mA g^−1^, ZMNO‐PBA maintains a specific capacity of 58.7 mAh g^−1^ after 200 cycles with a capacity retention rate of 74.6%. Ex situ XRD and XPS results demonstrate the minimal volumetric distortion during the charge–discharge process of ZMNO‐PBA with the Daniel‐type reaction mechanism. DFT calculations reveal the abnormal asymmetric nature of the diffusion path caused by lattice distortion significantly reduces the diffusion energy barrier for Na^+^ ions upon cycling. Besides, the well‐designed CNTT‐based dual‐salt electrolyte provides an extended voltage window and accelerates the solvation process of Mg^2+^ ions. The integrated strategy addresses the common challenges in structural regulation with cathode and dual‐ion electrolytes, which offers a new avenue for the development of Mg‐based hybrid batteries for high‐performance energy storage systems.

## 4 Experimental Section

### Synthesis of Cathode Materials

The ZMNO‐PBA was synthesized by a simple hydrothermal method. The precursor solutions include 20 mL of 0.034 m ZnCl_2_, 80 mL of 0.034 m MnCl_2_·4H_2_O, and 100 mL of 0.04 m Na_2_Fe(CN)_5_NO. An additional 0.876 and 3.504 g of sodium citrate acted as a reaction rate inhibitor was added to the precursor solutions of ZnCl_2_ and MnCl_2_·4H_2_O respectively to form solution A and B. Then 2 g ascorbic acid, 3 g PVP were added into 100 mL of 0.04 m Na_2_Fe(CN)_5_NO to form solution C. A mixture of A and B was added dropwise into C at a rate of 1 mL min^−1^ under continuous stirring. Finally, the solution was transferred into a Teflon autoclave and maintained at 110 °C for 3 h, then a blue precipitate was obtained. The synthesis procedure of ZM‐PBA was similar, just replace Na_2_Fe(CN)_5_NO with Na_4_Fe(CN)_6_, and then a white precipitate can be obtained. The product was washed two times with distilled water and washed once with acetone before drying at 80 °C under a vacuum oven overnight.

### Synthesis of Electrolyte CNTT

Firstly, 2 mL of DME was added into 0.5 m magnesium chloride, and stirred into a homogeneous solution, then 0.5 M aluminum chloride was added slowly, followed by 0.1 m magnesium bis(trifluoromethanesulfonic)imide, stirred for 3 h to get a clarified solution, and then 0.5 m sodium bis(trifluoromethanesulfonic)imide was added into the solution and stirred for 3 h to get a mixed electrolyte CNTT.

### Material Characterization

The crystal structure of ZMNO‐PBA was examined by XRD (Japan. Rigaku Ultima IV) and the elemental composition of ZMNO‐PBA and ZM‐PBA was analyzed by ICP (ICP‐OES, Agilent 5110). TG analysis (Netzsch TG 209 F1) was performed in the temperature range of 30–250 °C to evaluate the adsorbed and interstitial water content. Specific morphological features were observed by field emission scanning electron microscope (FESEM, JEOL, JSM‐6700F), and transmission electron microscopy (TEM, Tecnai G2F20 FEI). The transition of vibrational and rotational energy levels was tested by Fourier transform infrared spectroscopy (FTIR, Nicolet 380 FTIR spectrometer). Elemental chemical states were analyzed by X‐ray photoelectron spectroscopy (XPS, PHIESCA‐5000C).

### Electrochemical Characterization

The active substance, acetylene black and PVDF were mixed in a ratio of 7:2:1 to make a slurry, the mass of the active substance is ≈2 mg coated on a diameter of 10 mm molybdenum foil. The electrode was dried at 80 °C for 12 h under vacuum. A molybdenum two‐electrode mold cell was used for all electrochemical tests. The cells were assembled in a glove box filled with argon atmosphere (H_2_O and O_2_ < 0.1 ppm). The negative electrodes were made of magnesium foil polished under 1200‐grit sandpaper. Constant current charge/discharge tests at room temperature were performed using a Neware CT4008T with a voltage range of 0.7–3.25 V (vs Mg^2+^/Mg). The cyclic voltammetry was performed using an electrochemical workstation, a News chi660e, with a sweep rate of 0.1 mV s^−1^. The EIS (Electrochemical Impedance Spectroscopy) were carried out over the frequency range of 100 kHz to 0.01 Hz. The GITT (Galvanostatic Intermittent Titration Technique) test was conducted at a current density of 50 mAg^−1^ for 10 min, followed by a relaxation period of 40 min.

Equation (3) is used to determine the diffusion coefficient of active ions derived from Weppner et al., and the parameters needed can be obtained from GITT curves

(3) $$D_{Na^{+}}=\frac{4}{\pi \tau }{\left(\frac{m_{B}V_{M}}{M_{B}S}\right)}^{2}{\left(\frac{\Delta E_{s}}{\Delta E_{\tau }}\right)}^{2}\left(\tau \ll \frac{L^{2}}{D_{Na^{+}}}\right)$$

### Calculation Methodology

For the theoretical calculation, ZMNO‐PBA and ZM‐PBA were constructed based on the structure model of Fe_7_(CN)_18_. All the calculations were performed in the framework of the Density Functional Theory(DFT) with the projector augmented plane‐wave method, as implemented in the Vienna ab initio simulation package.^[^ ^51^ ^]^ The generalized gradient approximation proposed by Pardew, Burke, and Ernzerhof was selected for the exchange‐correlation potential.^[^ ^52^ ^]^ The DFT‐D3 correction was used to describe weak interactions between atoms.^[^ ^53^ ^]^ The cut‐off energy for the plane wave was set at 450 eV. The energy criterion was set at 10^−5^ eV in the iterative solution of the Kohn‐Sham equation. The Brillouin zone integration was performed using a 3 × 3 × 3 Monkhorst‐pack *k*‐point.^[^ ^54^ ^]^ All structures were relaxed until the maximum force on each atom to less than 0.02 eV Å^−1^.

The immigration path and the energy barriers of Na^+^ in were determined by the Climbing Image Nudged Elastic Band (CI‐NEB) method.^[^ ^55^ ^]^ The energy criterion was set at 10^−7^ eV in search of transitional states. All the images were relaxed until the residual forces on the atoms have declined to less than 0.03 eV Å^−1^.

## Conflict of Interest

The authors declare no conflict of interest.

## Supporting information

Supporting Information

## Data Availability

Research data are not shared.
