# Supplementary material for: A Novel Asymmetric Diffusion Path for Superior Ion Dynamic in High‐Voltage Mg‐Based Hybrid Batteries
Source: Adv Sci (Weinh). 2024 Sep 4;11(41):2406451. doi: 10.1002/advs.202406451 (PMC11538649; doi:10.1002/advs.202406451)
Supplement: Supplementary file 1 — Supporting Information [file ADVS-11-2406451-s001.docx]

Supporting Information

**A Novel** **Asymmetric Diffusion Path for Superior Ion Dynamic in High-Voltage Mg-based Hybrid Batteries**

Kaifeng Huang, Baihua Qu,* Xing Shen,* Rongrui Deng, Rong Li, Guangsheng Huang, Aitao Tang, Qian Li, Jingfeng Wang, Fusheng Pan*

**Table S1.** ICP results of ZMNO-PBA and ZM-PBA material’s molar ratio.

| Element | Na | Zn | Mn | Fe |
| --- | --- | --- | --- | --- |
| ZMNO-PBA | 6.62% | 6.79% | 3.53% | 39.79% |
| ZM-PBA | 8.41% | 4.61% | 12.91% | 14.31% |


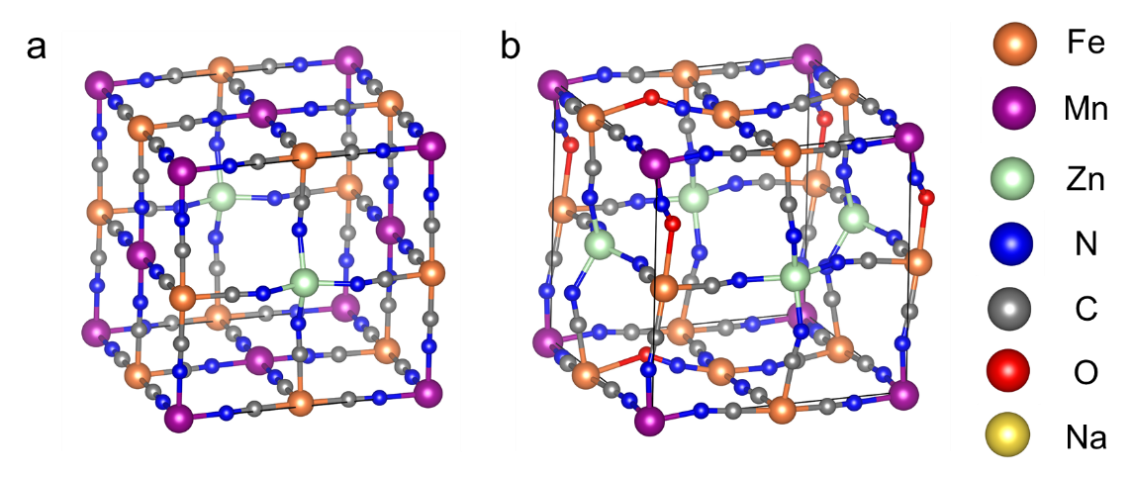


**Figure S1.** Side view of a) ZM-PBA and b) ZMNO-PBA crystal structures.

**Figure S2.** a) The LSV curve and b) CV curve of the CNTT electrolyte on Mo working electrode (10 mV s^-1^). c) Cycling performance of Mg|CNTT|Mg at 0.1 mA cm^–2^ with 0.1 mAh cm^–2^.

**Figure S3.** a) Electronic picture and b) cross-section SEM image of Magnesium anode after 60 cycles. c) EDS result and d-f) element mapping images of the Mg anode after 60 cycles in a hybrid cell at a current density of 200 mA g^−1^.

**Figure S4.** a) Rietveld refinement of ZM-PBA and b) the comparison of powder XRD patterns for ZM-PBA and ZMNO-PBA materials.

**Figure S5.** XPS spectrum of ZM-PBA: N 1s

**Figure S6.** SEM image of a) MnFe(CN)_5_NO and b) ZM-PBA. c) HAADF and d-i) Mapping images of ZM-PBA.

**Figure S7.** XPS analysis of ZMNO-PBA after initial discharge a) Mn 2p; b) Fe 2p

**** **Figure S8.** a) CV curves of ZM-PBA at a scan rate of 0.1 mV s^−1^. b) Charge-discharge curves at different current densities for ZMNO-PBA cathode. c) CV curves at scan rates of 0.2–1.0 mV s^−1^. d) Corresponding b-value.

**Figure S9.** GITT curves and corresponding diffusion coefficients upon charging and discharging for a) ZMNO-PBA and b) ZM-PBA.

**Figure S10.** Ex-situ XRD patterns of ZM-PBA at different charge/discharge states and the corresponding charging/discharging curves.

**Figure S11.** Side view of Na diffusion in a-c) ZM-PBA and d-e) ZMNO-PBA, including initial state, transitional state, and final state.

**Figure S12.** The COHP (Crystal Orbital Hamilton Population) curves of a) N=O, b) C≡N bond in ZMNO-PBA, c) C≡N bond in ZM-PBA.
